# Evaluation of Response to Immune Checkpoint Inhibitors Using a Radiomics, Lesion-Level Approach

**DOI:** 10.3390/cancers13236050

**Published:** 2021-12-01

**Authors:** Chorog Song, Hyunjin Park, Ho Yun Lee, Seunghak Lee, Joong Hyun Ahn, Se-Hoon Lee

**Affiliations:** 1 Department of Radiology and Center for Imaging Science, Samsung Medical Center, Sungkyunkwan University School of Medicine, Seoul 06351, Korea; chorog.song@samsung.com; 2 School of Electronic and Electrical Engineering, Sungkyunkwan University, Suwon 16419, Korea; hyunjinp@skku.edu; 3 Center for Neuroscience Imaging Research, Institute for Basic Science, Suwon 16419, Korea; 4 Department of Health Sciences and Technology, Samsung Advanced Institute for Health Sciences & Technology (SAIHST), Sungkyunkwan University, Seoul 06355, Korea; shlee119@skku.edu; 5 Core Research & Development Center, Korea University Ansan Hospital, Ansan 15355, Korea; seunghaklee@korea.ac.kr; 6 Biostatistics and Clinical Epidemiology Center, Samsung Biomedical Research Institute, Seoul 135710, Korea; jhguy.ahn@samsung.com; 7 Division of Hematology-Oncology, Department of Medicine, Samsung Medical Center, Sungkyunkwan University School of Medicine, Seoul 06351, Korea

**Keywords:** NSCLC, ICI, radiomics

## Abstract

**Simple Summary:**

Unique responses such as hyperprogressive disease (HPD) and a dissociated response (DR) have been reported after immune checkpoint inhibitor (ICI) treatment, and these patterns are difficult to evaluate with conventional methods. The aim of this study was to evaluate radiomics features of HPD at the lesion level, and to understand the clinical significance of a dissociated response. Our study revealed organ-specific radiomics features, likely reflecting the organ-specific microenvironment, that can be used to discriminate HPD. In addition, we observed that a dissociated response was associated with poor overall survival. A radiomic lesion-level approach shows great potential for response evaluation of ICI treatment.

**Abstract:**

Conventional methods to determine the response to immune checkpoint inhibitors (ICIs) are limited by the unique responses to an ICI. We performed a radiomics approach for all measurable lesions to identify radiomic variables that could distinguish hyperprogressive disease (HPD) on baseline CT scans and classify a dissociated response (DR). One hundred and ninety-six patients with advanced lung cancer, treated with ICI monotherapy, who underwent at least three CT scans, were retrospectively enrolled. For all 621 measurable lesions, HPDv was determined from baseline CT scans using the tumor growth kinetics (TGK) ratio, and radiomics features were extracted. Multivariable logistic regression analysis of radiomics features was performed to discriminate DR. Radiomics features that significantly discriminated HPDv on baseline CT differed according to organ. Of the 196 patients, 54 (27.6%) had a DR and 142 (72.4%) did not have a DR. Overall survival in the group with a DR was significantly inferior to that in the group without a DR (log rank test, *p* = 0.04). Our study shows that lesion-level analysis using radiomics features has great potential for discriminating HPDv and understanding heterogeneous tumor progression, including a DR, after ICI treatment.

## 1. Introduction

Lung cancer still has a poor prognosis, with the highest reported incidence and mortality among various malignancies [1]. Immune checkpoint inhibitors (ICIs) have recently been spotlighted for the treatment of lung cancer, as they improve outcomes, and have been rapidly adopted as a first-line treatment [2,3,4,5].

Conventional response assessment criteria such as the RECIST and WHO criteria are known to have limitations [6], and these limitations are even more pronounced when evaluating the response to ICI treatment due to the unique characteristics of ICIs [7]. Modified criteria such as the immune-related response criteria (irRC), immune-related RECIST (irRECIST), and modified RECIST 1.1 for immune-based therapeutics (termed iRECIST), have been proposed as alternatives [8,9,10], but these criteria only consider pseudoprogression [11].

In immunotherapy, a response pattern referred to as a dissociated response (DR) appears frequently; this pattern is characterized by the growth of some individual tumors and a decrease in size of others, which is clinically important and related to prognosis [12,13,14]. In addition, rapid disease progression may occur after the initiation of ICI, referred to as hyperprogressive disease (HPD), which has adverse clinical outcomes [15,16].

Since atypical response patterns such as DR and HPD refer to the response patterns of individual tumors, accurate understanding, consideration, and evaluation of these phenomena is possible only by using a lesion-level approach [17]. Using radiomic analysis, quantitative information can be extracted from medical images and analyzed to obtain a comprehensive understanding of the characteristics of individual tumors [18]. Recent studies have found that radiomic features have the potential to predict treatment response [19,20] and clinical outcomes [21,22,23,24].

Our aim in this study was to use a radiomics approach to determine if measurable lesions on baseline CT images of patients with stage IV non-small cell lung cancer (NSCLC) undergoing ICI had radiomic features consistent with HPD or not. In addition, we investigated the clinical potential of using lesion-level analysis to discriminate concordant/discordant responses.

## 2. Materials and Methods

Institutional review board (IRB 2021-03-024) approval was obtained for this retrospective study with a waiver of the requirement for informed consent.

### 2.1. Patients

**Figure 1:**
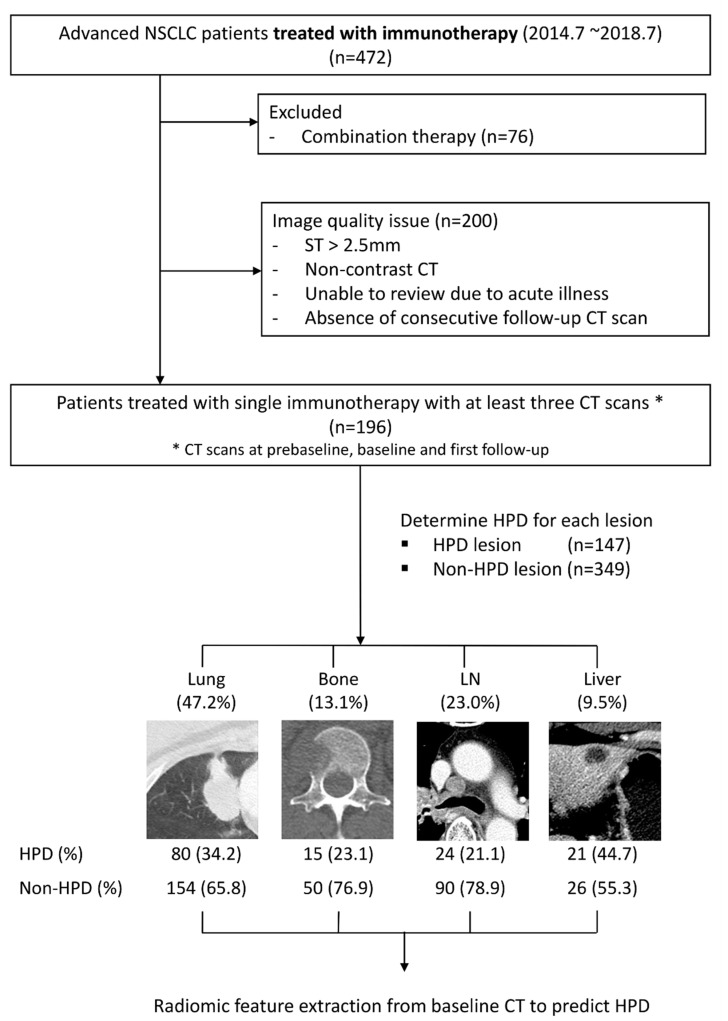
Flowchart of patient enrollment, exclusion, and our lesion-based radiomics approach. HPD, hyperprogressive disease; LN, lymph node; NSCLC, non-small cell lung cancer; ST, slice thickness.

We retrospectively identified patients with advanced NSCLC who received ICI treatment targeting either programmed death 1 (PD-1) or its ligand (PD-L1) between 1 July 2014 and 31 July 2018 at Samsung Medical Center. Only those patients who underwent ICI monotherapy were included. The exclusion criteria for CT imaging review were as follows: (a) slice thickness >2.5 mm, (b) non-contrast CT, (c) unable to review tumor status due to superimposed acute illness, and/or (d) absence of at least three consecutive CT scans (pre-baseline, baseline or follow-up) (Figure 1). Consequently, patients treated with single immunotherapy with at least three consecutive CT scans were included in our study.

### 2.2. Imaging

CT images were obtained with the following parameters: detector collimation, 1.25 or 0.625 mm; 120 kVp; 150–200 mA; and reconstruction interval 1–2.5 mm. All images were displayed at standard mediastinal (window width, 400 Hounsfield units [HU]; window level, 20 HU) and lung (window width, 1500 HU; window level, −700 HU) window settings. All CT scans were obtained after an injection of 80 cc of contrast material at 2 cc/s followed by a normal saline flush of 20 cc at 2 cc/s.

All measurable lesions per patient were included in the analyses. A lesion-based volumetry was performed for each lesion. Lesions with volumetry values were defined as index lesions, and for each index lesion, the region of interest was segmented using commercial software (Aview, version 1.0.23, 2018; Coreline Soft, Seoul, Korea) [25,26], and then radiomic features were extracted.

The HPD of index lesions was evaluated using the tumor growth kinetics (TGK) ratio. After determining the HPD using this formula, it was defined as HPDv lesion. The TGK ratio was calculated as the differences between tumor volumes from consecutive CT scans [27]. To overcome the limitations of RECIST when defining HPD, HPDv using volumetry was used. As a limitation of the RECIST criteria, first, the tumor burden cannot be fully reflected, and, in particular, there is a limitation that non-target lesions cannot be included. In a previous study, it was confirmed that this limitation could be improved with HPDv using the volume analysis of the entire lesion. HPDv was defined as follows: (a) time to treatment failure less than 2 months, (b) TGK ratio of 2 or more, and (3) volume increase of 50% compared with baseline [28].

To categorize dissociated and non-dissociated responses, the Nelson criteria based on volumetry values from lung nodule screening were used. Progression was defined when the volume of an individual index lesion increased by 25% or more, and a partial response was defined as a decrease of 25% or less in volume. Cases where lesions in different organs in one patient differed in progression and partial response were defined as DR cases. Patients with one target lesion were assigned to the non-DR group [29,30].

### 2.3. Radiomics Analysis

Lesions were grouped according to the organ involved, namely, lung, liver, lymph nodes, and bone. Radiomics feature extraction was performed on lesions visualized in baseline CT scans. Many small lesions were present in organs and we had to model many different organs; therefore, 19 histogram-based radiomics features and area features (2.5 D) for each organ were computed using an in-house MATLAB code (Mathworks Inc., Natick, MA, USA) and Pyradiomics [31]. Further details regarding the category and number of radiomics features adopted are provided in Table S5.

### 2.4. Statistical Analyses

Prior to statistical analyses, some features were log- or power- (1/2 and 1/3) transformed to compress the distribution of the features. For data that included negative numbers, cube conversion was performed.

We analyzed the 29 significant radiomics features, including transformed features (*n* = 14) and the original features (*n* = 20), to identify factors capable of discriminating HPDv using baseline CT data. The *p*-values of all 29 radiomics features from baseline CT scans were calculated using univariate generalized estimating equations (GEEs). Features with a *p*-value < 0.1 were selected as significant for HPDv discrimination.

Next, we analyzed the selected features using multiple GEEs. We performed subgroup analysis according to the involved organ. Furthermore, receiver operating characteristic (ROC) analysis with a calculation of the area under the receiver operating characteristic curve (AUC) was conducted to determine the sensitivity and specificity of various features with regard to their ability to discriminate HPDv.

The overall survival (OS) was calculated as the time interval between the first administration of an ICI and death due to any cause. Living patients were right-censored at the time of last contact. The Kaplan–Meier method was used to estimate the cumulative probability of overall survival of patients treated with immunotherapy. Differences between survival curves were analyzed with the log-rank test. Statistical analysis was performed using SPSS^®^ v17.0 for Windows (SPSS, Inc., Chicago, IL, USA) and the SAS^®^ software package (SAS Institute, Inc., Cary, NC, USA).

## 3. Results

### 3.1. Baseline Characteristics

A total of 472 patients, treated with ICIs between July 2014 and July 2018, were screened. Patients administered a combination of ICIs were excluded (*n* = 76). Patients with image quality issues were also excluded (*n* = 200) (Figure 1).

We analyzed 196 patients treated with ICI monotherapy for whom at least three CT scans were available. The best overall responses assessed by RECIST 1.1 were partial response (PR) in 31 patients (15.8%), stable disease (SD) in 24 patients (12.2%), and progressive disease (PD) in 141 patients (71.9%).

### 3.2. Determination of HPDv at the Lesion Level

We extracted the tumor volume for each lesion and examined variations in TGK for 621 lesions. Among the 621 lesions, there were 147 HPDv lesions (23.7%) and 349 non-HPDv lesions (56.2%). HPDv is defined as growth that is more than double that of tumor growth before the ICI treatment [28]. Therefore, in order to determine HPDv, it is necessary to assume that the lesion must be present in all three images: pre-baseline, baseline, and first follow-up. Therefore, 125 lesions that were not in the pre-baseline, or disappeared at the first follow-up, were excluded.

The numbers of HPDv lesions in the lungs, bone, lymph nodes, and liver were 80 (54.4%), 15 (10.2%), 24 (16.3%), and 21 (14.3%), respectively. The number of non-HPDv lesions in the lungs, bone, lymph nodes, and liver were 154 (44.1%), 50 (14.3%), 90 (25.8%), and 26 (7.4%), respectively. There was no significant difference in clinicopathologic variables between HPDv and non-HPDv lesions (Tables S1 and S2).

### 3.3. Univariate and Multivariate Analyses

Univariate analyses of 29 variables identified 15 variables that were significantly associated with HPDv. In the subgroup analysis by organ, there were nine variables for the lungs, four variables for the bone, nine variables for the lymph nodes, and one for the liver. These statistically significant variables are listed in Table S3.

Table 1 shows the results of the multivariate analysis of radiomic features. In the lung, the significant radiomic variables were Log (Uniformity_HIST*1000) (*p* = 0.001, OR = 0.29) and Log(Volume) (*p* = 0.002, OR = 0.71), and the area under the receiver operating characteristic curve (AUC) value of these two was 0.65 (*p* value< 0.002). In the bone, Log (Uniformity_HIST * 1000) (*p* = 0.017, OR = 4.49) was a significant variable with an AUC of 0.70. In the lymph nodes and liver, Log(RMS) (*p* = 0.025, OR = 3.88) with an AUC of 0.63 and Percentile histogram 2.5 (Cube-root transformation) (*p* = 0.006, OR = 0.74) with an AUC of 0.72 were significant variables, respectively.

### 3.4. Prognosis Analysis

**Figure 2:**
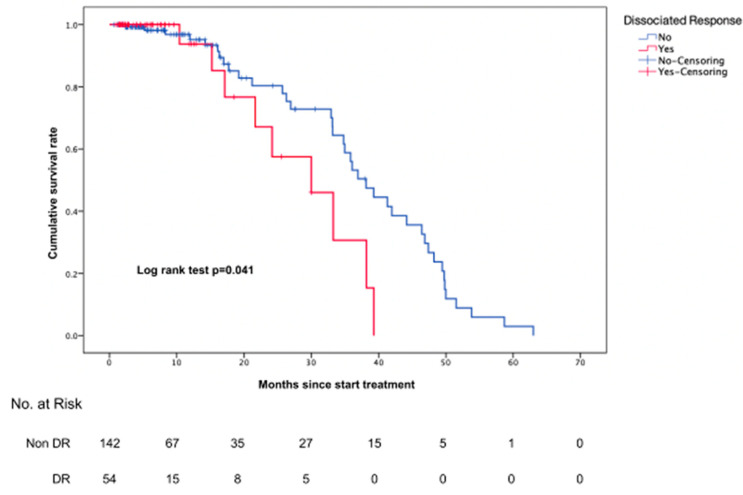
Comparison of overall survival between patients with a DR and those without a DR.

Of the 196 patients, 54 (27.6%) had a DR and 142 (72.4%) did not have a DR (Table S4). OS in the group with a DR was significantly inferior to that in the group without a DR (log rank test, *p* = 0.04) (Figure 2).

In the analysis according to response group, there was a significant difference in the overall survival, especially in the PR and SD groups (Figure S1).

## 4. Discussion

To evaluate the treatment response to ICIs, we used a lesion-level radiomics approach. We found heterogeneous ICI responses for each organ based on lesion-level radiomic features extracted from baseline CT scans. The characteristic radiomics features of lesions that were classified as HPDv differed according to organ location. Concordant and discordant responses to ICIs were classified by integrating the response evaluation for each lesion and patients with a DR showed poor outcomes.

In recent studies, heterogeneous responses from organs to ICI have been observed and metastatic liver lesions have been reported to have a particularly poor response [17,32]. Similarly, in one study, patients with liver lesions had a low overall survival rate [20]. These heterogeneous responses to ICI can be partially explained by differences in the organ-specific tumor microenvironment [33,34], as the mechanism of ICI therapy is based on the effect of ICI on tumor infiltrating lymphocytes (TILs), and other immune cells in the tumor microenvironment [35].

**Figure 3:**
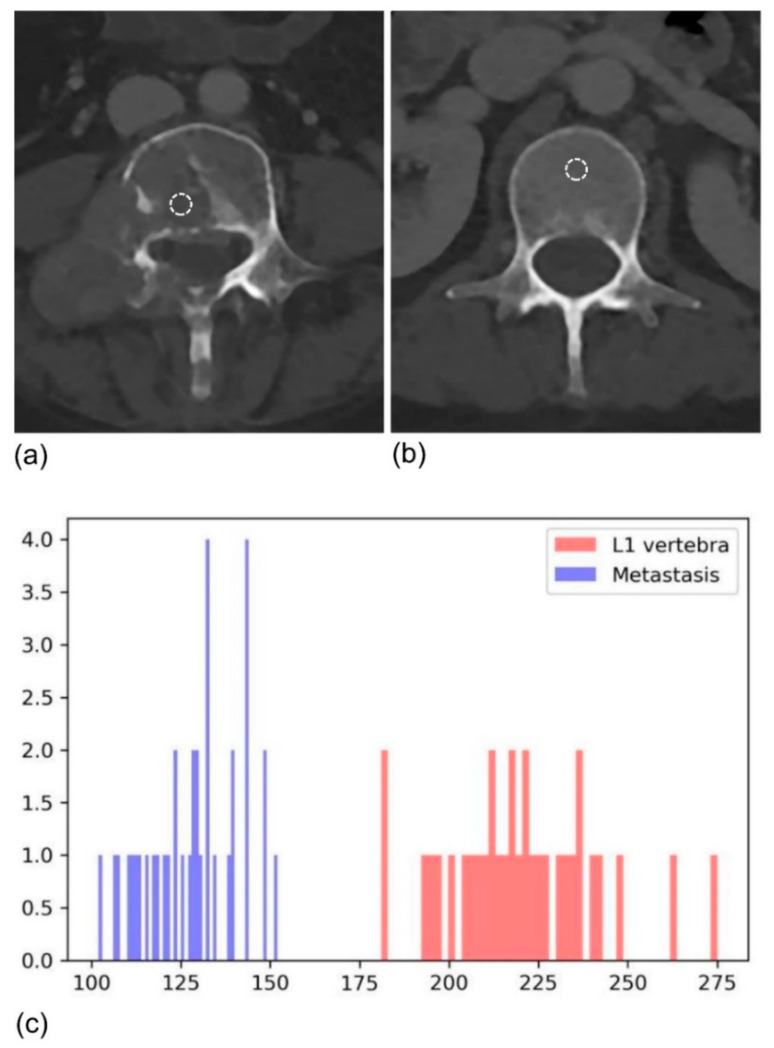
Baseline CT of a 47-year-old female with NSCLC showing (**a**) metastasis and (**b**) the normal L1 vertebra as a reference. (**c**) The uniformity of metastasis was higher than that of the normal reference, meaning that metastasis-related voxels were homogenous.

When evaluating treatment response, it would be helpful to use a lesion-level radiomics approach to assess the unique behavior of ICI treatment. We used quantitative data extracted from baseline CT images for HPDv discrimination. Different radiomics variables were selected for different organs, likely reflecting the unique characteristics of each organ. Curiously, in both the lung and bone, Log(Uniformity_HIST*1000) was selected with an opposite OR direction (0.29 in lung, 4.49 in bone). Uniformity indicates the homogeneity of voxels, and we found a correlation between heterogeneous lung lesions and HPD progression. This result is consistent with the association between high tumor heterogeneity and the poor outcomes reported in previous studies [21,36]. Conversely, in the bone, the uniformity_HIST value of the metastatic lesion was 0.054, whereas that of the reference normal vertebra was 0.035, indicating that the metastatic lesion was rather homogenous (Figure 3). Tumor cells disrupt the normal bone remodeling process, causing the disappearance of the normal bone matrix [37,38]. CT images show mainly radiolucent and osteolytic lesions and are thought to be more homogenous than normal bone tissue [39]. Meanwhile, in the lymph nodes, Log (RMS) was selected, which reflects the magnitude of the histogram. A recent study by Coroller et al. showed that radiomic features indicating a sphericity of the primary tumor and lymph node homogeneity were correlated with better pathologic responses in NSCLC patients with concurrent chemoradiation therapy [40], which is consistent with our findings. In the case of the liver, Percentile histogram 2.5 (Cube-root transformation) was a significant variable. This variable measures the intensity values at the 2.5th percentile on the histogram and indicates that lesions with lower CT attenuation showed hyperprogression in the liver. Hepatic metastasis is frequently accompanied by necrosis, which is caused by tumor hypoxia and mainly occurs in centers with poor blood and oxygen supply [41,42]. In addition, the genomic stability of tumor cells in the tumor center is much lower, with a higher possibility of distant metastasis [43]. It is thought that necrosis of the tumor center further promotes tumor cell mutation, and that this is related to HPD.

The clinical impact of a DR is unclear. In one previous study, a DR was shown to be to be associated with an unfavorable prognosis [14]. However, recent studies have reported better overall survival than true progression in patients with a DR [12,44]. In our study, patients with a DR had poor overall survival (Figure 2). When response groups were compared, significant differences were seen in the PR and SD groups but not the progression group (Figure S1). DR is associated with a poor prognosis in response group patients; therefore, it can be used as an important factor in determining whether to maintain treatment in this group. Thus, further research on response criteria, including a DR, is needed.

In addition, the process of obtaining volume data using volumetry does not require additional advanced imaging techniques. CT images already taken for treatment in daily practice are used without additional imaging or the application of other imaging techniques. If only a semiautomated tumor segmentation is performed, then the volume can be obtained by calculation using computer software, which is possible through a commercialized program or even an open-source program [45,46,47,48]. Therefore, lesion-level evaluation through volumetry is expected to have great clinical potential as well as not being difficult to apply to actual daily practice.

The limitations of our study include its single-center nature, the small sample size, and the sample from a single site. To overcome this weakness, additional external validation is needed in future multi-center large cohort studies. Next, using a lesion-level approach, it was impossible to assess for both new and disappeared lesions on follow-up which may have resulted in selection bias. However, we used an ROI segmentation and radiomics approach for all lesions present on the CT scans collected at three points in time.

## 5. Conclusions

Organ-specific radiomics features from baseline CT scans were useful for HPDv discrimination. A dissociated response was classified by integrating the findings of the response evaluation for each lesion, and a DR was shown to be correlated with a poor outcome. As a preliminary study on the usefulness of a lesion-level radiomics approach, our findings highlight the potential for using this type of approach to capture intra- and inter-tumor heterogeneity.

## Figures and Tables

**Table 1:** Multivariate analysis of radiomics data according to organ.

Organ	Variable	*p*-Value	OR	AUC (95% CI)
All	Kurtosis_HIST	0.030	0.98 (0.96, 0.99)	0.61 (0.55–0.66)
	Percentile histogram 2.5 (Cube-root transformation)	0.012	0.94 (0.89, 0.98)	
Lung	Log(Uniformity_HIST*1000)	0.001	0.29 (0.13, 0.61)	0.65 (0.58–0.72)
	Log(Volume)	0.002	0.71 (0.58, 0.88)	
Bone	Log(Uniformity_HIST*1000)	0.017	4.49 (1.31, 15.37)	0.70 (0.56–0.85)
Lymph nodes	Log(RMS)	0.025	3.88 (1.18, 12.70)	0.63 (0.49–0.77)
Liver	Percentile histogram 2.5 (Cube-root transformation)	0.006	0.74 (0.60, 0.91)	0.72 (0.57–0.88)
Others	Median_HIST	0.025	1.05 (1.00, 1.09)	0.70 (0.48–0.92)

## Data Availability

The data presented in this study are available in this article and Supplementary Materials.

## Supplementary Materials

The following are available online at https://www.mdpi.com/article/10.3390/cancers13236050/s1, Figure S1: Comparison of overall survival between patients with a DR and patients without a DR. Subgroup analysis was performed by best overall response (as assessed by RECIST 1.1), Table S1: Baseline characteristics of study patients and CT, Table S2: Radiomics features of non-HPDv and HPDv lesions, Table S3: Univariate analysis of radiomics features to discriminate HPDv, Table S4: Determination of dissociated response and classification according to best response group, Table S5: Definition of extracted radiomics features.

## Abbreviations

AUC	area under the receiver operating characteristic curve
DR	dissociated response
GEE	generalized estimating equation
HPD	hyperprogressive disease
ICI	immune checkpoint inhibitor
iRECIST	modified RECIST 1.1 for immune-based therapeutics
irRC	immune-related response criteria
irRECIST	immune-related RECIST
NSCLC	non-small cell lung cancer
OS	overall survival
PD	progressive disease
PD-1	receptor programmed cell death 1
PD-L1	programmed death ligand 1
PR	partial response
ROC	receiver operating characteristic
SD	stable disease
TGK	tumor growth kinetics
