# Supplementary material for: Evaluation of Response to Immune Checkpoint Inhibitors Using a Radiomics, Lesion-Level Approach
Source: Cancers (Basel). 2021 Dec 1;13(23):6050. doi: 10.3390/cancers13236050 (PMC8657103; doi:10.3390/cancers13236050)
Supplement: Supplementary file 1 [file cancers-13-06050-s001.zip › cancers-1459325-supplementary.pdf]

Supplementary Materials

Table S1. Baseline characteristics of study patients and CT scans.

Characteristics	HPD (n=58, 29.6%)	nonHPD-PD (n=83, 42.3%)	SD (n=24, 12.2%)	PR (n=31, 15.8%)	P value
Median age, y (range)	60 (34-81)	59 (33-84)	60 (37-78)	59 (40-74)	0.736
Sex, (M/F)	44/14	54/29	21/3	22/9	0.154
Smoking history, (%)					0.420
Never-smoker	27	45	9	13	
Ever-smoker	31	38	15	18	
Histologic subtype (%)					0.155
ADC	36	65	15	24	
SQCC	21	17	8	6	
LCNEC	1	1	1	1	
ECOG PS, (%)					0.011
0 or 1	39	60	13	27	
≥ 2	19	23	11	4	
No. of prior therapies					0.259
< 3	12	11	4	9	
≥ 3	46	72	20	22	
PD-L1 status (%)					0.642
PD-L1 ≥ 1%	25	41	14	15	
PD-L1 < 1%	14	21	4	5	
Unknown	19	21	6	11	

HPD, hyperprogressive disease; ADC, adenocarcinoma; SQCC, squamous cell carcinoma; LCNEC, large cell neuroendocrine carcinoma; ECOG PS, Eastern Cooperative Oncology Group performance status; PD-L1, programmed death ligand 1

Table S2. Radiomics features of non-HPDv and HPDv lesions.

Characteristics	Non-HPDv (n=349)	HPDv (n=147)	P value
Volume (mean [SD])	61277.70 (124354.03)	42133.03 (139922.57)	0.132
Max_HIST (mean [SD])	518.74 (475.10)	435.69 (449.71)	0.072
Min_HIST (mean [SD])	-547.96 (391.05)	-573.09 (392.50)	0.514
Median_HIST (mean [SD])	46.85 (168.52)	-8.37 (207.69)	0.002
mean_HIST (mean [SD])	33.73 (178.92)	-25.36 (209.82)	0.002
var_HIST (mean [SD])	18818.36 (25582.01)	24056.28 (28338.69)	0.044
Energy_HIST (mean [SD])	1013167649.17 (2327445108.74)	670911025.33 (1413683512.15)	0.098
std_HIST (mean [SD])	112.01 (79.32)	128.81 (86.69)	0.037
Skewness_HIST (mean [SD])	-0.96 (1.74)	-0.75 (1.52)	0.195
Kurtosis_HIST (mean [SD])	13.52 (19.80)	8.92 (13.07)	0.01
RMS_HIST (mean [SD])	176.26 (144.33)	206.17 (161.43)	0.043
IQR_HIST (mean [SD])	132.11 (124.78)	157.17 (136.50)	0.048
Range_HIST (mean [SD])	1066.70 (658.53)	1008.78 (638.52)	0.367
Entropy_HIST (mean [SD])	7.86 (1.05)	7.96 (1.04)	0.293
Uniformity_HIST (mean [SD])	0.01 (0.01)	0.01 (0.01)	0.324
PH2_5 (mean [SD])	-205.95 (293.17)	-307.72 (345.33)	0.001
PH25 (mean [SD])	-25.99 (205.54)	-94.68 (254.18)	0.002
PH50 (mean [SD])	46.85 (168.52)	-8.37 (207.69)	0.002
PH75 (mean [SD])	106.12 (166.78)	62.49 (180.05)	0.01
PH97_5 (mean [SD])	205.08 (214.63)	161.96 (187.65)	0.035
TGK_ratio (mean [SD])	-26.72 (380.69)	59.00 (453.52)	0.031

Table S3. Univariate analysis of radiomics features to discriminate HPDv.

Organ	Results of univariate analysis			
	Variable	p-value	OR	AUC
**Total**	Median_HIST	0.00266	0.998 (0.997, 0.999)	0.558 (0.501, 0.615)
	Mean_HIST	0.00234	0.998 (0.997, 0.999)	0.565 (0.508, 0.622)
	Kurtosis_HIST	0.0257	0.981 (0.965, 0.998)	0.582 (0.528, 0.636)
	PH2_5	1.44E-03	0.999 (0.998, 1.000)	0.575 (0.518, 0.632)
	PH25	0.00308	0.999 (0.998, 1.000)	0.564 (0.507, 0.621)
	PH50	0.00266	0.998 (0.997, 0.999)	0.558 (0.501, 0.615)
	PH75	0.00973	0.998 (0.997, 1.000)	0.560 (0.503, 0.618)
	PH97_5	0.0366	0.999 (0.998, 1.000)	0.550 (0.493, 0.606)
	log_Kurtosis	0.00857	0.752 (0.609, 0.929)	0.582 (0.528, 0.636)
	Cube_PH2_5	0.0128	0.939 (0.893, 0.987)	0.575 (0.518, 0.632)
	Cube_PH25	0.00506	0.941 (0.901, 0.982)	0.564 (0.507, 0.621)
	Cube_PH50	0.00306	0.913 (0.870, 0.959)	0.558 (0.501, 0.615)
	Cube_PH75	0.00274	0.932 (0.889, 0.976)	0.560 (0.503, 0.618)
	Cube_PH97_5	0.0306	0.922 (0.875, 0.973)	0.550 (0.493, 0.606)
	log_Volume	0.0138	0.890 (0.811, 0.976)	0.579 (0.524, 0.633)
**Lung**	Kurtosis_HIST	0.0385	0.972 (0.947, 0.998)	0.611 (0.537, 0.686)
	RMS_HIST	0.0415	1.002 (1.000, 1.004)	0.594 (0.517, 0.671)
	log_Kurtosis	0.0072	0.690 (0.527, 0.904)	0.611 (0.537, 0.686)
	log_RMS	0.0257	1.745 (1.069, 2.849)	0.594 (0.517, 0.671)
	log_IQR	0.0473	1.467 (1.005, 2.141)	0.588 (0.510, 0.665)
	log_Uniformity	0.0356	0.600 (0.373, 0.966)	0.593 (0.514, 0.671)
	logUniformity_HIST1000	0.0356	0.600 (0.373, 0.966)	0.593 (0.514, 0.671)
	Volume	0.0175	1.000 (1.000, 1,000)	0.601 (0.529, 0.674)
	Log_Volume	0.0468	0.900 (0.812, 0.998)	0.601 (0.529, 0.674)
**Bone**	Entropy_HIST	0.0128	0.320 (0.130, 0.785)	0.729 (0.582, 0.877)
	log_Energy	0.0177	0.616 (0.413, 0.919)	0.680 (0.512, 0.848)
	log_Uniformity	0.0167	4.482 (1.309, 15.346)	0.709 (0.560, 0.858)
	logUniformity_HIST1000	0.0167	4.482 (1.309, 15.346)	0.709 (0.560, 0.858)
**LN**	Median_HIST	0.0209	0.997 (0.994, 1.000)	0.467 (0.330, 0.604)
	mean_HIST	0.0139	0.996 (0.993, 0.999)	0.509 (0.371, 0.648)
	var_HIST	0.0441	1.000 (1.000, 1.000)	0.538 (0.400, 0.676)
	PH2_5	0.000644	0.996 (0.994, 0.998)	0.557 (0.42, 0.694)
	PH25	0.0206	0.997 (0.994, 1.000)	0.585 (0.460, 0.709)
	PH50	0.0209	0.997 (0.994, 1.000)	0.467 (0.330, 0.604)
	PH75	0.0268	0.997 (0.994, 1.000)	0.517 (0.374, 0.660)
	PH97_5	0.0441	0.996 (0.993, 1.000)	0.556 (0.419, 0.692)
	log_Max	0.0445	0.393 (0.158, 0.977)	0.560 (0.425, 0.696)
**Liver**	cube_PH2_5	0.0054	0.742 (0.600, 0.919)	0.728 (0.575, 0.881)
**Other**	Median_HIST	0.0182	1.037 (1.006, 1.069)	0.685 (0.465, 0.905)
	PH50	0.0182	1.037 (1.006, 1.069)	0.685 (0.465, 0.905)
	Cube_PH50	0.0305	7.316 (1.210, 44.225)	0.685 (0.465, 0.905)
	Volume	0.0439	1.000 (1.000, 1.000)	0.635 (0.382, 0.889)

Table S4. Determination of dissociated response and classification according to response group.

	**Dissociated response**		
	Yes (n=54, 27.6%)	No (n=142, 72.4%)	Total (n=196)
**HPD**	24 (41.4%)	34 (58.6%)	58
**Non-HPD PD**	20 (24.1%)	63 (75.9%)	83
**SD**	4 (16.0%)	21 (84.0%)	25
**PR**	6 (20.0%)	24 (80.0%)	30

HPD, hyperprogressive disease; PD, progressive disease; SD, stable disease; PR, partial response

Table S5. Definition of extracted radiomics features.

	Parameter	Formula	Description
**Histogram-based features** [1]	Max, Min	$\text{Max} = Max(X(i))$ or $\text{Min} = Min(X(i))$ where X denotes the 3d image matrix with N voxels	Measures maximum or minimum intensity value of a histogram
	Median	$\text{Median} = \frac{X(i)}{2}$ where X denote the 3D image matrix	Measures median intensity value of a histogram
	Mean	$\text{Mean} = \frac{1}{N}\sum_{i}^{N} X(i)$ where X denotes the 3D image matrix with N voxels	Measures mean intensity value of a histogram
	Variance	$\text{Variance} = \frac{1}{N-1}\sum_{i=1}^{N}(X(i)-\bar{x})^2$	Measures squared distances of each value of a histogram from the mean
	Standard deviation	$\text{Std} = \left(\frac{1}{N-1}\sum_{i=1}^{N}(X(i)-\bar{x})^2\right)^{1/2}$ where X denote the 3D image matrix with N voxels	Measures amount of variation of a histogram.
	Energy	$\text{Energy} = \sum_{i}^{N} X(i)^2$ where X denotes the 3D image matrix with N voxels	Measures squared magnitude value of a histogram
	Skewness	$\text{Skewness} = \frac{E(x-\mu)^3}{\sigma^3}$ where μ is the mean of x, σ is the standard deviation of x, and E is the expectation operator	Measures asymmetry of a histogram.
	Kurtosis	$\text{Kurtosis} = \frac{E(x-\mu)^4}{\sigma^4}$ where μ is the mean of x, σ is the standard deviation of x, and E is the expectation operator.	Measures “peakedeness” of a histogram (flatness of histogram)
	Root mean square (RMS)	$\text{RMS} = \sqrt{\frac{1}{N}\sum_{n=1}^{N}\lvert X_n\rvert^2}$ where X denotes the 3D image matrix with N voxels	Measures the square-root of the mean of the squares of the values of the histogram. This feature is another measure of the magnitude of a histogram

	Interquartile range	$\mathrm{IQR} = \mathrm{Q}_3 - Q_1$<br>where Q_3 denotes the 3rd quartile of the histogram and Q_1 denotes the 1st quartile of the histogram	Measures of variability, based on dividing a histogram into quartiles
	Range	$\mathrm{Range} = \mathrm{range}(\mathrm{X(i)})$	Measures difference between the highest and lowest voxel values of a histogram
	Percentile	$\mathrm{Percentile} = \left(\frac{n^{th}\ percentile}{100}\right) \mathrm{X(i)}$	Measures intensity value at the 2.5th, 25th ,50th ,75th, and 97.5th percentiles on a histogram
	Entropy	$\mathrm{Entropy} = -\sum_{i=1}^{N_l} P(i) \log_2 P(i)$<br>where P denotes the first-order histogram with N_l discrete intensity levels	Measures the irregularity of a histogram
	Uniformity	$\mathrm{Uniformity} = \sum_{i=1}^{N_l} P(i)^2$<br>where P denotes the first-order histogram with N_l discrete intensity levels	Measures uniformity of a histogram
**Size-based features** [1, 2]	Area	$\mathrm{Area} = \mathrm{R} * \text{number of voxels}$<br>where R denote the ROI resolution	Area of the tumor (ROI)

1. Aerts HJWL, Velazquez ER, Leijenaar RT, et al (2014) Decoding tumour phenotype by noninvasive imaging using a quantitative radiomics approach. Nat Commun 5:4006. https://doi.org/10.1038/ncomms5006

2. Aerts HJWL, Grossmann P, Tan Y, et al (2016) Defining a Radiomic Response Phenotype: A Pilot Study using targeted therapy in NSCLC. Sci Rep 6:. https://doi.org/10.1038/srep33860

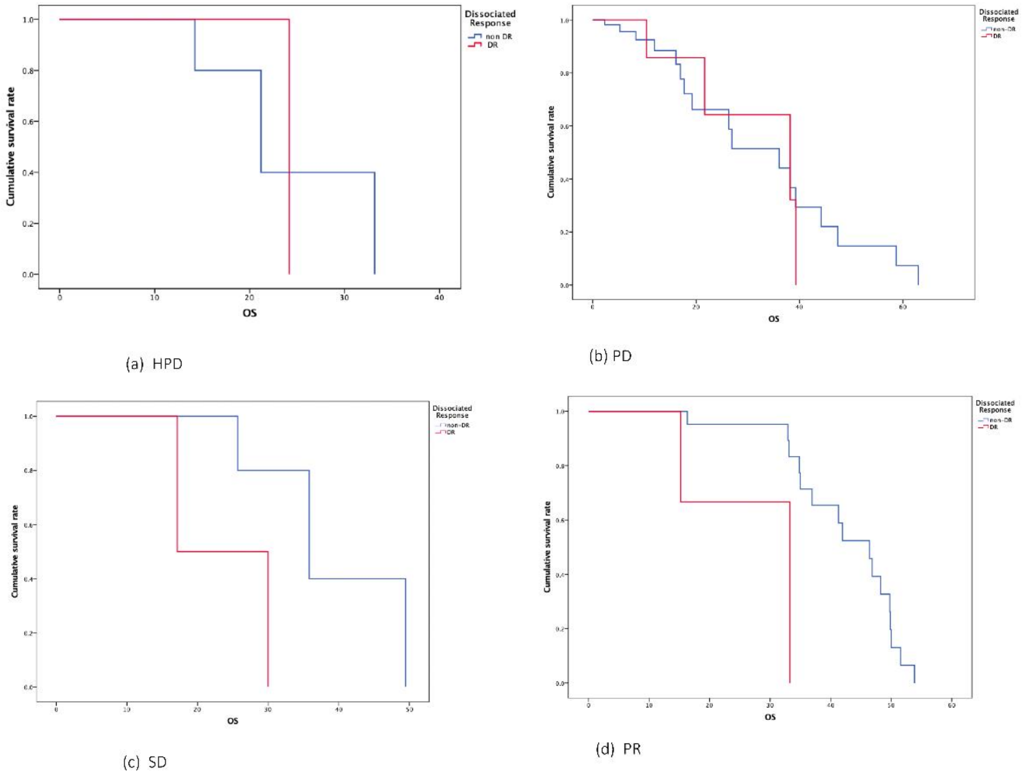

Figure S1. Comparison of overall survival between patients with a DR and patients without a DR, and subgroup analysis by best overall response (as assessed by RECIST 1.1). (a) HPD, (b) PD, (c) SD, (d) PR.

DR, dissociated response; HPD, hyperprogressive disease; PD, progressive disease; SD, stable disease; PR, partial response
